# *Salmonella spp*. quorum sensing: an overview from environmental persistence to host cell invasion

**DOI:** 10.3934/microbiol.2021015

**Published:** 2021-06-24

**Authors:** Amanova Sholpan, Alexandre Lamas, Alberto Cepeda, Carlos Manuel Franco

**Affiliations:** 1 Almaty Technological University, Almaty, Republic of Kazakhstan; 2 Universidade de Santiago de Compostela, Lugo, Spain

**Keywords:** *Salmonella*, quorum sensing, environment, persistence, host cell invasion

## Abstract

*Salmonella* spp. is one of the main foodborne pathogens around the world. It has a cyclic lifestyle that combines host colonization with survival outside the host, implying that *Salmonella* has to adapt to different conditions rapidly in order to survive. One of these environments outside the host is the food production chain. In this environment, this foodborne pathogen has to adapt to different stress conditions such as acidic environments, nutrient limitation, desiccation, or biocides. One of the mechanisms used by *Salmonella* to survive under such conditions is biofilm formation. Quorum sensing plays an important role in the production of biofilms composed of cells from the same microorganism or from different species. It is also important in terms of food spoilage and regulates the pathogenicity and invasiveness of *Salmonella* by regulating *Salmonella* pathogenicity islands and flagella. Therefore, in this review, we will discuss the genetic mechanism involved in *Salmonella* quorum sensing, paying special attention to small RNAs and their post-regulatory activity in quorum sensing. We will further discuss the importance of this cell-to-cell communication mechanism in the persistence and spoilage of *Salmonella* in the food chain environment and the importance in the communication with microorganisms from different species. Subsequently, we will focus on the role of quorum sensing to regulate the virulence and invasion of host cells by *Salmonella* and on the interaction between *Salmonella* and other microbial species. This review offers an overview of the importance of quorum sensing in the *Salmonella* lifestyle.

## 1. Introduction

*Salmonella spp*. is a genus of facultative anaerobe Gram-negative bacilli with flagella and motility, composed of 2,659 different serotypes according to the last supplement to the White-Kauffmann-Le Minor scheme [1]. This genus only contains two species, *Salmonella bongori and Salmonella enterica*. The latter is composed of six subspecies, namely *enterica* (I), *salamae* (II), *arizonae* (IIIa), *diarizonae* (IIIb), *houtenae* (IV), and *indica* (VI) [1]. *Salmonella* is one of the main foodborne pathogens worldwide, together with *Campylobacter spp*. In 2018, *Salmonella* was responsible for 91,857 confirmed cases of human salmonellosis in the European Union (EU). The species *S. enterica* subsp. *enterica* and, precisely, *S*. Typhimurium and *S*. Enteritidis, are the main species responsible for human infections [2]. *Salmonella* can be isolated from a wide range of animals and their products, including poultry, bovines, ovine, porcine, fish, seafood, lizards, snakes, or crocodiles. *Salmonella enterica* subsp. *enterica* is mainly related to warm-blooded animals, whereas the other subspecies of *S. enterica* subsp. *enterica* are closely related to cold-blooded animals [3]. Of those, poultry products are the main source of human salmonellosis [4]. To move forward through the food chain, *Salmonella* must be resistant to different environmental stress conditions such as heat, desiccation, nutrient starvation, or biocides. This implies that this pathogen must rapidly adapt to different environmental conditions in order to survive, and this adaptation is regulated by a complex genetic machinery activating metabolic pathways associated with stress resistance [5]; in these pathways, small RNAs play an important regulatory role [6]–[8]. To adapt to these stressful situations, bacterial cells have mechanisms that help them act together towards a common goal. One of the main mechanisms that allow bacterial cells to act together is quorum sensing [9], which can be defined as a cell-to-cell communication tool that controls different processes such as luminescence, sporulation, virulence, or biofilm formation. Also, quorum sensing can be extended to inter-kingdom communication through extracellular signaling molecules, so-called ‘autoinducers’ [10]. It is important to understand the mechanisms of quorum sensing from two angles. On the one hand, to learn about the mechanisms that bacteria use to communicate with each other and with the host. On the other hand, a full understanding of the functioning of these mechanisms allows the development of quorum quenching methods to inhibit the growth of pathogenic bacteria [11]. This review will evaluate the main quorum sensing mechanisms used by the genus *Salmonella* and its importance in the food chain.

## 2. Quorum sensing mechanism

### 2.1. Definition of quorum sensing

The first signs of quorum sensing were observed at the end of the 1960s and in the early 1970s, when it was found that genetic competence in *Streptococcus pneumoniae* [12] and luminescence production by two marine bacteria were regulated by the production of extracellular molecules [12],[13]. However, these discoveries passed unnoticed for almost 20 years [14]. In the 1980s, the discovery of the luminescence (*lux*) genes of the marine bacterium *Vibrio fischeri* and the genes that regulate the expression of lux gene, luxI and luxR implied a great step forward in the quorum sensing field [15],[16]. Also, it was determined that the quorum sensing signal from *V. fischeri* was N-3-oxohexanoyl-L-homoserine lactone (3OC6-HSL) [17]. Thus, luxI codes the synthase required for 3OC6-HSL synthesis, and luxS codes the 3OC6-HSL-responsive transcriptional activator of the lux genes. In the 1990s, DNA sequencing and the comparative sequence analysis revolution allowed to discover gene pairs with homology to luxR and luxI, indicating that this mechanism is present in a huge range of microorganisms [14].

Fuqua et al. [18] introduced the term quorum sensing to define bacterial cell-to-cell communication. Through this mechanism, bacteria can communicate with cells of their own species and of other species by small, diffusible molecules called autoinducers. This mechanism is used by bacteria to detect modifications in their surrounding environment, with the aim to apply specific strategies for adaptation to environmental stress in space and time [19]. Thus, bacteria use quorum sensing for different purposes, such as bioluminescence, enhancing access to nutrients or environmental niches, defensive responses against eukaryotic hosts and competing organisms, cell differentiation into morphological forms (biofilm cells, sporulation), or survival in growth-restrictive environments [20]. The signal molecules are secreted at a basal level during bacterial growth and accumulate in the environment. The accumulation of quorum sensing molecules can indicate a high population density or a low-density population that has been producing quorum sensing molecules for a long period of time [21]. When the concentration of quorum sensing molecules reaches a threshold level (the quorum level), this causes changes in gene expression in receptor cells through transcription factors that detect the quorum sensing molecules. In the case of intraspecies communication, the emitter and the responder are usually the same cells. On some occasions, the genes involved in the synthesis of quorum sensing molecules and their receptors activate their own expression without any external intervention, which explains the term autoinducers [13].

### 2.2. Mechanism of quorum sensing

**Figure 1.**
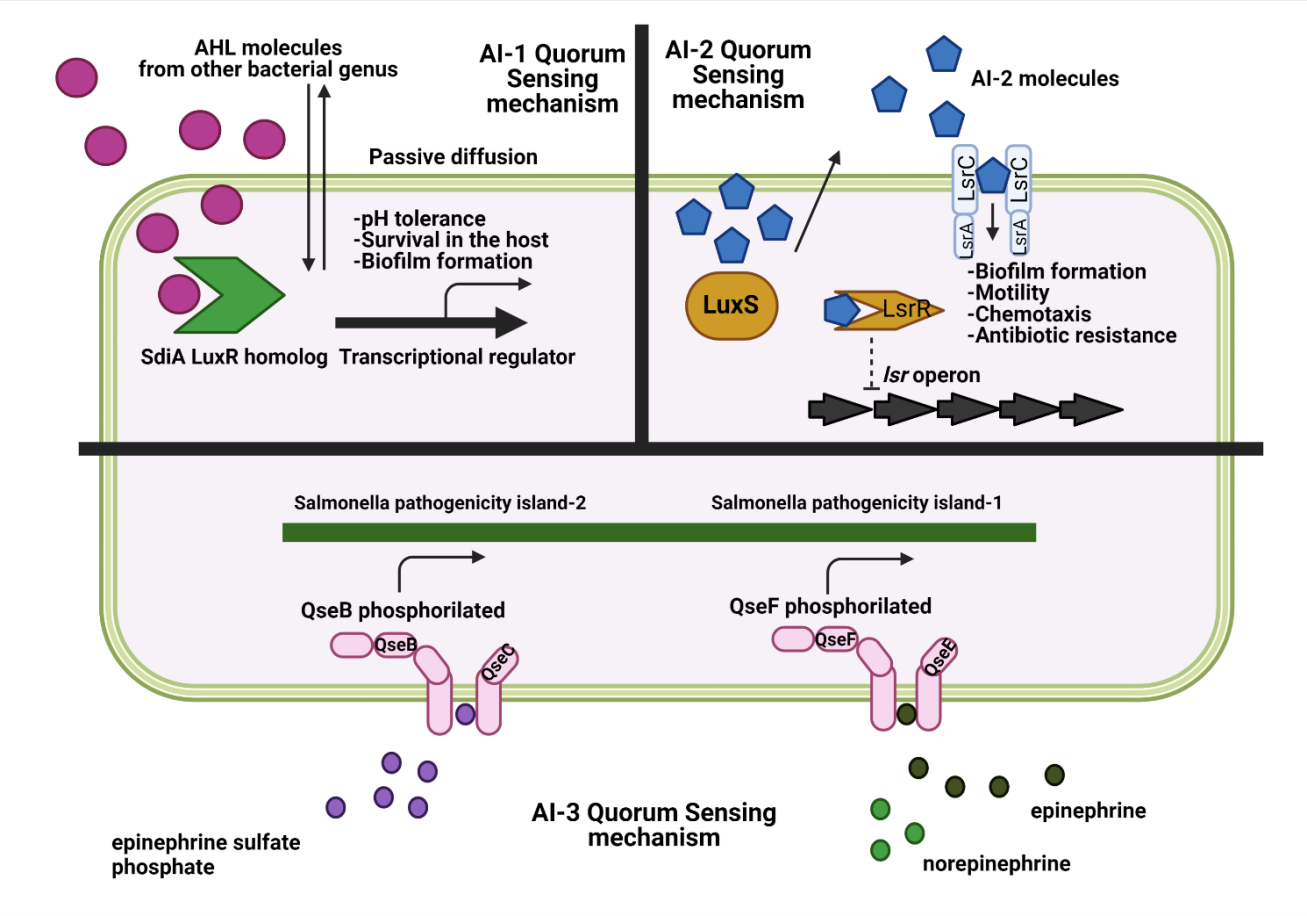
Overview of Salmonella quorum sensing mechanisms and the main bacterial routes regulated by each of these mechanisms. Created with biorender.com.

The signaling compounds used by cell-to-cell communication in bacteria can be divided into four different categories. The first are the fatty acid derivatives N-acyl homoserine lactones (AHLs), generally called autoinducer-1 (AI-1), that are produced and used by Gram-negative bacteria mainly for intraspecies communication [22]. The second compounds are furanosyl borate diesters derived from the recycling of S-adenosyl-homocysteine to homocysteine, also called autoinducer-2 (AI-2). These molecules are produced by both Gram-positive and Gram-negative bacteria and assumed to serve as universal signals for interspecies and intraspecies communication [23]. The third compound is the autoinducer-3 (AI-3), whose function is the cross-talking with mammalian epinephrine host cell signaling systems [24] (Figure 1). Finally, Gram-positive bacteria use peptides or modified peptides (autoinducing peptides) for cell-to-cell communication. These peptides are characterized by their small size (6–25 amino acid residues), stability, specificity, and diversity. They are synthetized as precursor peptides in ribosomes, processed to active mature autoinducer signal molecules, and secreted by an ATP-binding cassette transporter. Peptides exert their function intercellularly or extracellularly, depending on the location of the sensor [25].

#### 2.2.1. AI-1

**Figure 2.**
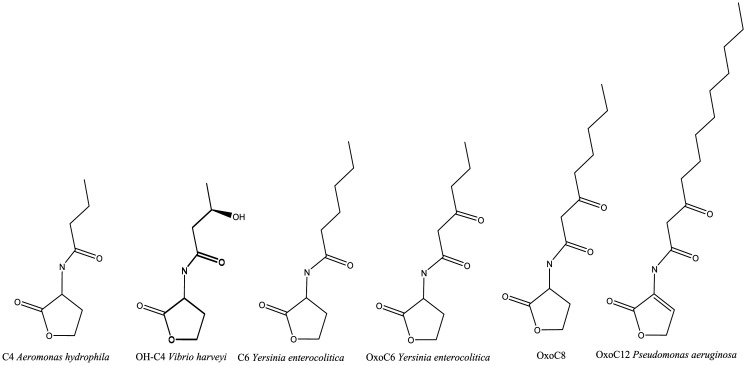
AHLs structures produced by different microorganism.

The LuxIR system is the prototypical quorum sensing system and based on the production and detection of AHLs by *V. fischeri*. The gene luxI codes the AHL signal molecule, and the gene luxR codes the transcription factor that detects the presence of AHL. The formed LuxR-AHL complex activates the transcription of the luxICDABE operon, required for bioluminescence in *V. fischeri* [26],[27]. Since the discovery of AHLs in *V. fischeri*, homologs of this mechanism have been observed in most Gram-negative bacteria. The different AHL variants synthesized by the different LuxR homologues have an acyl chain length of 4 to 18 carbon atoms (Figure 2). The chain also presents variations in saturation, with most of the chain being saturated, with some exceptions. In addition, the three positions of the acyl chain can be unmodified or present a carbonyl or hydroxyl group (oxo). These differences in the AHLs and their receptors indicate some degree of species specificity in AI-1.

**Figure 3.**
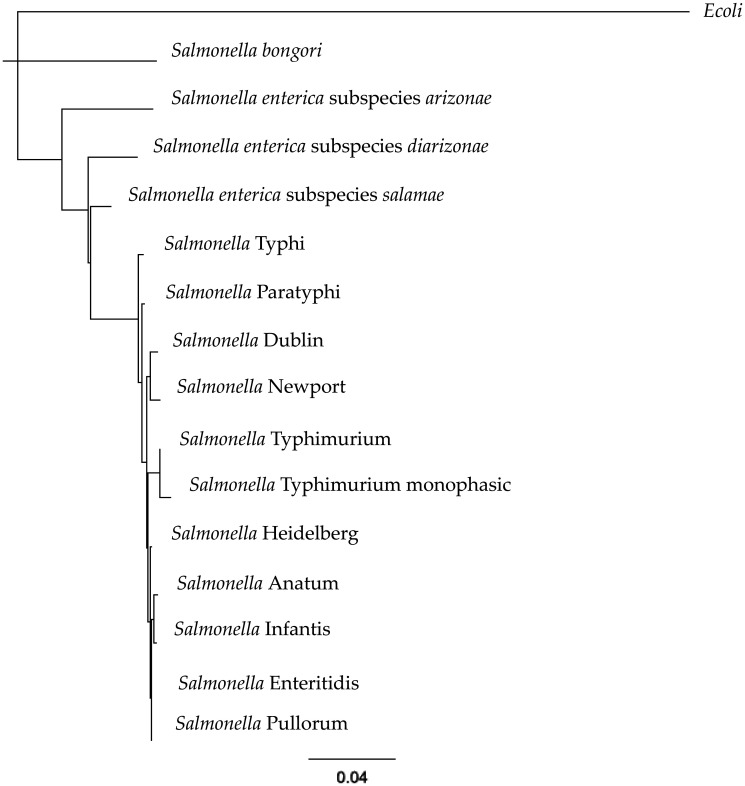
Phylogenetic tree elaborated with the sdiA sequences of *Escherichia coli* and the sequences of the different species and subspecies of *S. enterica*.

However, the case of *Salmonella* and other bacterial genera, such as *Escherichia*, *Klebsiella, Enterobacter*, or *Citrobacter* is special. These bacteria encode a LuxR homolog called sdiA, an AI-1 receptor, but do not synthetize AHLs because the gene necessary for synthesis, LuxI, is not encoded in their genome. As a result, SdiA is considered an orphan or solo LuxR homolog [28],[29]. The gene that encodes SdiA follows a parallel evolution with bacterial genera. *Escherichia coli* and *Salmonella* diverged of a common ancestor 120–150 million years ago. Also, *S. bongori* and *S. enterica* diverged 40–63 million years ago. *Salmonella enterica* subspcies *arizonae* was the first subspecies to diverge, whereas the subspecies *indica* and *salamae* were the last to diverge, about 20 million years ago [30]–[32]. Figure 3 shows a phylogenetic tree elaborated with the *sdiA* sequences of *Escherichia coli* and the sequences of the different species and subspecies of *S. enterica*. It is possible to observe how the S. *bongori* sequence diverged of S. *enterica*. In the same sense, the remaining *S. enterica* subspecies showed different distances, with the subspecies *arizonae* showing the higher distance to the subspecies *enterica*.

Although AHLs are, in contrast to AI-2, considered an intraspecies communication system, the lack of the *luxI* homolog in *Salmonella* indicates that this system can also be used for interspecies communication and may have great importance in *Salmonella* host gut colonization [33]. In this sense, *Salmonella* SdiA protein can detect a wide range of AHLs produced by other bacterial species, with preference for the modified structure oxoC8. However, *Salmonella* can detect AHLs with other structures, such as C4 and oxoC12, produced by *Pseudomonas aeruginosa*, the C4 produced by *Aeromonas hydrophila*, and the C6 and oxoC6 produced by *Yersinia enterocolitica* [33],[34].

As mentioned before, *Salmonella* is composed of more than 2,600 different serotypes. Also, *S. enterica* is divided into six subspecies that have gained and/or lost different virulence determinants during their evolution [30]. Therefore, the response of the different serotypes to quorum sensing signals can be different by the regulation of different genes. In the specific case of *S*. Typhimurium, the *sdiA* gene regulates two specific loci, namely the *rck* operon and the *srgE* gene. The *rcK* operon is located in the 95k-b virulence plasmid pSLT, and the operon is composed of the six genes *pefI*, *srgD*, *srgA*, *srgB*, *rck*, and *srgC*. Genes *pefI*, *srgD*, and *srgC* encode transcription factors that exert regulatory effects on the *pef* operon of pSLT. This operon codifies Pef fimbriae, whose function is adhesion to crypt epithelial cells, induction of proinflammatory responses, and biofilm formation [35]. In the same way, *srgA* contributes to the folding of the PefA fimbrial subunit and the SsaC protein, coded in *Salmonella* Pathogenicity Island 2 (SPI-2), and is part of the type III secretion system [36]. Another gene of this operon is *rck*, which codifies an outer membrane protein involved in resistance to complement killing and adhesion to fibronectin and laminin. It is remarkable that *rck* operon is not expressed at temperatures below 37 °C, which highlights the importance of this operon in *S*. Typhimurium virulence and the close relationship between quorum sensing and virulence regulation. Finally, gene *srgE* is a single-gene horizontal acquisition in the chromosome and related to type III-secreted effector [37]–[39].

#### 2.2.2. AI-2

**Figure 4.**
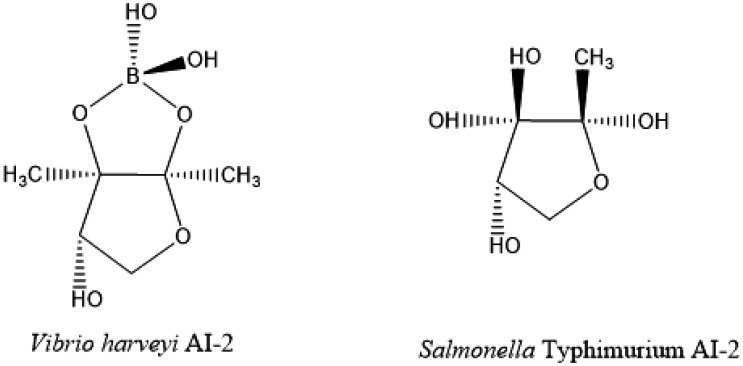
Structures of AI-2 synthetized by *Vibrio harvey* and *Salmonella* Typhimurium.

Twenty years ago, it has been observed that *S*. Typhimurium and *E. coli* produced and secreted a small, soluble, heat-labile signaling molecule called autoinducer-2 [40]. Some years before, the presence of a second quorum sensing system in bacteria had been proposed [41]. Thus, it was observed that an AHL-blind reporter strain was able to produce bioluminescence only upon induction of the AI-2-dependent second system. The most interesting fact was that this strain responded to culture fluids from several unrelated bacteria (Figure 4). These data indicated that the production of AI-2 was not restricted to a single bacterial species and that its production could be extended through bacteria [41]. The presence of homologues of the gene luxS, responsible for AI-2 activity, has been observed in several sequence genomes [42], and every *luxS*-containing species could be correlated with the detection of AI-2 activity in the extracellular media, suggesting that bacterial cells use AI-2 to communicate with cells of other bacterial species [40],[41].

In *S*. Typhimurium, the production of AI-2 is linked to the exponential growth phase, with a maximum concentration at the mid-exponential phase. At the beginning of the stationary phase, this signal starts to disappear from the media, along with glucose depletion [43]. Based on a previous study, AI-2-dependent signaling requires low pH and high osmolality, as low osmolality induces signal degradation [40]. The production of AI-2 in *S*. Typhimurium requires a series of enzymatic reactions. In the first place, S-adenosyl methionine is converted to S-adenosylhomocysteine by methyl transferases, which is then converted to 4,5 dihydroxy-2,3-pentanedione by methylthioadenosine/S-adenosylhomocysteine nucleosidase (Pfs) and S-rybosylhomocysteinase (LuxS). In the final step, 4,5 dihydroxy-2,3-pentanedione is cyclized into (2R,4S)-2-methyl-2,3,3,4-tetrahydroxytetra-hydrofuran (AI-2). As mentioned before, the production of AI-2 in *S*. Typhimurium is related to exponential growth. Once AI-2 is synthetized, the signaling molecule is extracellularly transported by the transport protein YdgG. Then, extracellularly located AI-2 binds to autoinducer-binding protein LsrB and is transported into the bacterial cell via an ATP transporter encoded on the *lsr* operon. Once inside the *Salmonella* cell, AI-2 is phosphorylated by LsrK and inactivates the transcription repressor protein LsrR in a dose-dependent manner, inducing the transcription of operon *lsrACDBFGE*. Also, LsrR inhibits the expression of some genes related to oxidate stress responses (*sodA*, *sodCI*, *sodCII*) with high importance for survival inside macrophages and represses flagella expression and virulence. Therefore, the inactivation of LsrR by AI-2 seems to be necessary for SPI-1 transcription and flagella expression. Phosphorylated AI-2 is degraded by LsrG and LsrF [43]–[46].

#### 2.2.3. AI-3

The last quorum sensing system currently known is autoinducer-3 (AI-3). However, until now, the synthetic pathway and chemical formula of AI-3 remain unknown. It is interesting that the two component regulators associated with AI-3 are also associated with the recognition of epinephrine and norepinephrine, two catecholamines produced by the host [47],[48]. Thus, *S*. Typhimurium encodes orthologs of the *E. coli* two-component regulatory systems QseC/B and QseF/E. While QseC can sense AI-3, epinephrine, and norepinephrine, QseE can also sense sulfate and phosphate [49]. After binding to AI-3/epinephrine/norepinephrine, QseC is autophosphorylated, causing the dephosphorylation of the response regulator QseB. This results in the transcription of SPI-2 genes. Also, the two-component regulatory system QseC/QseB causes modifications in the transcription of genes related with motility, invasion, and survival in macrophages [50]. While QseC/QseB is related to systemic disease and replication in macrophages, the other two-component regulatory system QseF/E exerts its regulatory function during epithelial cell invasion [49]. It has been demonstrated that catecholamines induce growth in iron-restricted media, induce the expression of virulence genes located in SPI-1 and SPI-3, and modulate the virulence of *S*. Typhimurium both *in vitro* and *in vivo*. This regulation may be vinculated to QseC/E [50]. It is necessary to mention that the α-adrenergic antagonist can block the QseC-dependent signaling, and some studies have shown that those antagonists can inhibit the motility enhanced by catecholamines [51],[52]. Finally, microarray studies have found that the catecholamines epinephrine and norepinephrine reduce the expression of the AI-2 *lsr* operon in *E. coli*. Therefore, it is possible that catecholamines cause a negative virulence regulation of genes vinculated to AI-2, most likely because of the different roles of AI-2 and AI-3 in the different steps of bacterial invasion of host cells. The importance of AI-3 in the human host is a promising field to elucidate the interactions between bacteria and their hosts.

### 2.3. The role of small RNAs in quorum sensing

In the last decade, small RNA (sRNA) discovery in microorganisms has revolutionized the world of microbiological research. These molecules are stable, abundant, and relatively short transcripts composed of 50–400 nucleotides. They primarily act as post-transcriptional regulators and carry out their activation or repression activity in two general ways: sRNA-protein interaction or direct sRNA-mRNA pairing [53],[54]. The use of sRNAs is a highly useful and immediate mechanism for regulating gene expression due to their rapid synthesis and low energy cost. There are two main categories of sRNAs described so far: trans-encoded sRNAs and cis-encoded sRNAs. Trans-encoded sRNAs are the most studied so far in bacteria and act by base-pairing with the target transcripts through imperfect and limited complementarity, resulting in altered stability and translation. These characteristics of *trans*-encoded sRNAs indicate that they can control a wide number of genes and, thus, exert regulatory functions in a wide range of regular processes such as metabolic regulation and stress resistance. Because the binding between trans-encoded sRNA and mRNA takes place through imperfect pairing, it requires the Hfq RNA chaperone to facilitate and stabilize this binding [55]. On the other hand, *cis*-encoded sRNAs are characterized by a region perfectly complementary to the target transcript. These types of sRNAs have initially been identified as regulators of mobile elements, including plasmids, phages, and transposons [56].

Although the role of small RNAs in *Salmonella* quorum sensing regulation is still unknown, studies in other bacterial groups have provided an idea of the importance of this post-transcriptional mechanism. In the early 21st century, it was observed that small RNA chaperone Hfq and multiple small RNAs control quorum sensing in *Vibrio cholerae*. The Hfq mediates interactions between small RNAs and the mRNA of the *V. cholerae* quorum-sensing master regulator HapR, which is implicated in virulence and biofilm formation [57],[58]. In *V. cholerae*, there are four redundant sRNAs (Qrr1-4) that regulate the quorum sensing signal transduction pathway; sRNA expression is based on cell population density. The HapR activates the expression of these four sRNAs that repress the translation of mhapR. Therefore, these sRNAs are involved in a negative feedback loop that causes the transition out of the quorum-sensing mode [59]. *Vibrio harveyi* showed a similar behavior, with five sRNAs (Qrr1-5) that destabilize the mRNA of LuxR, master regulator of quorum sensing. The LuxR directly activates the expression of those sRNAs that mediate a negative feedback loop that controls quorum sensing [60],[61]. In the case of *P. aeruginosa*, some sRNAs implicated in quorum sensing regulation have also been identified. It has been observed that RsmY and RsmZ sRNAs regulate the synthesis of the quorum sensing signal N-butanoyl-homoserine lactone [62], and another study found that sRNA P27 regulates the *P. aeruginosa* RhlI-RhlR quorum sensing system by binding to rhlI mRNA in a Hfq-dependent manner, affecting rhamnolipid production and biofilm formation [63]. In another study, it has been observed that *P. aeruginosa* PhrD sRNA increased RhlR transcript levels [64]. This demonstrates the complex regulatory network in which sRNAs are involved. In a bacterial species such as *E. coli*, phylogenetically related to *Salmonella*, some preliminary studies on the role of sRNAs in quorum sensing have also been performed. The LsrR, a protein involved in the network for the uptake of *E. coli* autoinducer 2 (AI-2), affected the generation of some sRNAs, including DsrA, with impacts on biofilm formation, among other things [65]. Although these articles did not evaluate *Salmonella*, they do provide clues to the fundamental role played by sRNAs in the regulation of quorum sensing. Undoubtedly, one of the great challenges of microbiology is to unravel the whole network of sRNAs present in bacteria and their regulatory functions. In the case of quorum sensing, it could be useful to develop new bacterial inhibition methods.

## 3. Quorum sensing in the food chain

Quorum sensing is a tool that allows *Salmonella* to adapt to different stress conditions in the food chain, with different food preservation techniques. One of them is the use of modified-atmosphere packaging; one strategy is to reduce or completely eliminate the levels of oxygen and replace it with other gases that favor the preservation of food products [66]. An *in vitro* study has shown that microaerobiosis and anaerobiosis reduced the expression of quorum sensing-related genes in comparison to aerobiosis in *S. enterica* isolated from poultry houses. As a result, *Salmonella* produced less biofilm in conditions with low levels of oxygen [67]. As previously commented, *Salmonella* has an incomplete AI-1 system as only synthetized the receptor. A study with *S*. Enteritidis found that the external addition of the AI-1 N-dodecanoyl-DL-homoserine lactone (C12-HSL) to the growth medium increased biofilm formation on the polystyrene surface [68]. *Salmonella* can optimize its metabolism in the presence of these AHLs [69]. Therefore, in packaged food, *Salmonella* could respond to the production of AI-1 by the natural microbiota of the food and increase biofilm formation.

The production of a biofilm by foodborne pathogens is a major concern for industry and food safety. Biofilms are structured bacterial cell communities enclosed in self-produced polymeric matrices that can adhere to abiotic or biotic surfaces [70]. In the case of *Salmonella*, cellulose and curli fimbriae are the two principal components of the polymeric matrix [71]. Biofilms increase the resistance of *Salmonella* cells to adverse conditions such as acidic environments, low temperature, heat, antimicrobials, and different atmospheric conditions [72],[73]. Biofilm formation is influenced by external and internal factors and regulated by a complex network of genes and small RNAs playing a key role in quorum sensing [73]. Quorum sensing seems to be important in all steps of biofilm formation, from initial adhesion to biofilm dispersion. It regulates cell density population, transcription profile, and metabolic activity. During biofilm formation, bacterial cells have to adapt from free-living planktonic cells to biofilm cells, which requires a coordinated gene expression to adapt to a new lifestyle. Thus, the response to quorum sensing-signaling molecules by bacterial cells allows to coordinate the production of adhesion and extracellular polymeric substances. It is important not to consider biofilms as a collection of cells of the same strain, as they are usually formed by bacteria of different genera. Therefore, the recognition of quorum sensing molecules produced by other bacterial genera is essential in the first steps of biofilm formation [74]. For example, Bai and Rai tested the effects of AHLs C4-HSL and C6-HSL at a concentration of 1 µM in *Salmonella* biofilm formation and observed that these molecules increased the attachment of *Salmonella* cells and the production of exopolymeric substances [75].

A singular study evaluated the role of (AHLs) produced by *Hafnia alvei* on *S*. Typhimurium biofilm formation in stainless-steel and polystyrene surfaces [76], showing that the quorum sensing molecule produced by this ubiquitous microorganism did not influence *S*. Typhimurium biofilm formation. It is therefore possible that *Salmonella* may not be able to recognize AHLs produced by certain bacteria or that it may do so under conditions other than those tested in this study. Another study [77] tested the effects of different AHL molecules on *S*. Enteritidis biofilm formation and gene expression. The results obtained showed that AHL C12-HSL had the higher influence, increasing biofilm formation and the expression of biofilm- and virulence-related genes under anaerobiosis. In a similar study, Dorou et al. [78] evaluated the effect of quorum sensing signals of cell-free supernatant (CFS) of *P. aeruginosa*, *Yersinia enterocolitica*-like GTE 112, *Serratia proteamaculans* 00612, *Y. enterocolitica* CITY650, and *Y. enterocolitica* CITY844 on the growth of *S*. Enteritidis and *S*. Typhimurium. While *P. aeruginosa* CFS increased the metabolic activity of *Salmonella* strains, the other CFS had the opposite effect. However, this study used CFS instead of purified quorum sensing molecules. As a consequence, it is possible that other compounds present in CFS also influence the growth of *Salmonella*, limiting the results of this study. A preliminary study evaluated the influence of quorum sensing in other stress conditions observed in the food-processing chain, such as heat and acid. The results of the study found no association between the resistance of *S*. Typhimurium and *S*. Thompson to these conditions and the production of AI-2 [79]. Quorum-sensing controls bacteria realease from mature biofilm [80], which is essential for colonizing new niches. The extracellular polymeric matrix present in mature biofilm protects *Salmonella* cells from external aggression but impedes bacterial release. High-density populations in biofilm activate the quorum sensing mechanism, which in turn can switch off the production of polysaccharide intracellular adhesin and activate the production of short peptides with detergent activity. Therefore, the quorum sensing mechanism would be key for bacterial release [81].

Because of the importance of the quorum sensing mechanism in the persistence of *Salmonella* in the food production chain, it is crucial to look for alternatives that block this mechanism to facilitate the eradication of this food-borne pathogen. Almasoud et al. [82] found that *Salmonella* can produce AI-2 in cantaloupe homogenates, and the addition of lactic and malic acid could effectively inhibit the quorum sensing activity. Similarly, Amrutha [83] also found promising results with lactic acid and acetic acid, as well as minor activity of citric acid. Conversely, the presence of AHLs can increase the resistance of *Salmonella* to natural antimicrobials such as nisin [84]. Essential oils are also a promising alternative to inhibit quorum sensing activity. For example, cumin (*Cuminum cyminum*) and pepper (*Piper nigrum*) oil nano-emulsions showed the capacity to reduce biofilm formation in *S. enterica* by 39%, and cumin could reduce quorum sensing activity in the indicator bacterium *C. violaceum* by 42% [85]. In a similar study, *Allium sativum* and *C. cyminum* essential oils reduced the expression of the quorum sensing-related genes *sdiA* and *luxS* and cellulose synthesis genes (*csgD* and *adrA*) [86]. By-products of the food industry have also been explored as a source of antimicrobial and anti-quorum sensing molecules. For example, Li et al. [87] tested punicalagin, an essential component of pomegranate rind, and observed a potential antimicrobial activity of this compound. Punicalagin inhibited quorum sensing in the sensor bacteria *C. violaceum* and downregulated the quorum sensing-related genes *sdiA* and *srgE* in *Salmonella*. Antimicrobial peptides can also be used as quorum sensing inhibitors. For example, Ma et al. [88] engineered a short β-sheet peptide WK2 interference with autoinducer-2 (AI-2)-mediated quorum sensing.

## 4. Quorum sensing in host colonization and virulence

Species of the genus *Salmonella* use quorum sensing to successfully colonize complex environments such as the gut [43]. In this environment, there are significant concentrations of bacterial autoinducers. Although some autoinducers are used for intraspecies communication, the autoinducer AI-2, synthetized by LuxS, acts as a universal signal that allows communication among species [40]. This autoinducer plays a key role in the regulation of niche-specific behavior such as biofilm formation, cell division, motility, and virulence in commensal and pathogenic bacteria [89]. Thus, quorum sensing modulates the expression of virulence genes in *Salmonella*. Microarray analysis has been used to determine the genes regulated by AI-2 in *S*. Typhimurium, using a wild strain and its Δ*luxS* mutant. In the mid-log phase of growth, there were 547 genes differentially expressed in the mutant strain. The genes were related with different cellular processes such as metabolism, motility, biofilm formation, transcription, and translation. In addition, biofilm formation and motility were altered in the mutant strain. Although the mutant strain showed a downregulation of flagellar motility, chemotaxis, and SPI-1 related genes, the expression of *hilD*, *hilA*, *sipB*, and *invABCE* virulence factors increased [90], demonstrating the complex mechanism of virulence regulation exerted by quorum sensing. Choi et al. [91] observed that a mutant strain with depletion of the *luxS* gene downregulated the expression of *invE* and other genes encoded in the SPI-1. The addition of the synthetic molecule or a plasmid encoding the *invE* gene restored the normal activity of the strain. The protein LsrR negatively controls invasiveness and motility of *Salmonella* through regulation of SPI-1, and its overexpression impaired the invasion of epithelial cells by *Salmonella*. The autoinducer AI-2 inactivates LsrR, resulting in an overexpression of SPI-1 genes [90]. Nesse et al. evaluated the role of N-acylhomoserine lactone quorum sensing signals in the invasion potential of *S*. Typhimurium and found that the invasion of epithelial cells by a wild *S*. Typhimurium strain was enhanced by the addition of C6-AHL and C8-AHL to the growth media in comparison to mutant strains lacking the gene *sdiA*, which encodes a receptor of AI-1 molecules. Also, the addition of these molecules increased the expression of the SdiA-regulating genes *rck* and *srgE* [92]. In this sense, SdiA directly regulates the *pefI-srgC* operon encoding the Rck invasion and modulates the invasiveness of *Salmonella* [93]. *Salmonella*. Typhimurium *luxS* mutants showed poor growth in nutrient-limited medium M9. However, the external addition of AI-2 restored the growth of this mutant strain, but it could not restore its ability to invade and survive in macrophages. However, the combination of AI-2 with long-chain fatty acids restored macrophage invasion, suggesting that a close relationship between nutrient availability and virulence regulation and quorum sensing alone is not sufficient to activate virulence [94]. In an *in vivo* study, SdiA was activated during the transit of *S. enterica* through the gastrointestinal tract of turtles which was colonized by the AHL-producing species *Aeromonas hydrophila*. Therefore, this study demonstrated that *Salmonella* can respond to the presence of AHL *in vivo* [95]. The use of *Galleria mellonella* as an infection model demonstrated that the presence of N-dodecanoyl-homoserine lactone increased the virulence of *S*. Enteritidis and increased persistence in hemolymph and in the hemocytes [96]. In this sense, SdiA is an important modulator of *Salmonella* virulence.

Quorum sensing is also an important modulator of the gut microbiota. Thompson et al. [97] observed that *E. coli* with increased synthesis of AI-2 modifies the composition of antibiotic-induced gut microbiota dysbiosis. The presence of this bacterium increased the levels of Firmicutes and the ratio of Firmicutes/Bacteroidetes in contraposition of the effects of antibiotics that caused Firmicutes clearance. This result also demonstrated that AI-2 is an important modulator of the major phyla of gut microbiota. In an innovative study, Ismail et al. [98] found that the intestinal epithelium produces an AI-2 mimic. This molecule is detected by the AI-2 receptor of bacteria, activating quorum sensing-related genes. The researchers postulate that this AI-2 mimic production could be stimulated by some bacterial mechanisms, demonstrating cross-kingdom signaling between host and bacteria and its potential role in gut symbioses. It has been proposed that quorum quenching can be used to modulate gut microbiota. The use of a native AI-2 kinase produced by *E. coli* phosphorylated AI-2 and reduced the quorum sensing response [99]. These strategies could be used in the future to modulate gut microbiota.

As previously discussed, bacteria are also capable of responding to molecules produced by the host, specifically to stress-related molecules such as catecholamines [100]. One of the first studies in 2007 showed that the growth of *Salmonella* is influenced by the presence of catecholamines, especially dopamine and norepinephrine [101]. An *in vivo* study found that the oral administration of norepinephrine to pigs increased the shedding of *Salmonella* [102]. Another study also observed that the administration of dopamine to mice infected with *S*. Typhimurium increased the loads of this bacteria in the liver and spleen and reduced mice survival through the stimulation of bacterial iron incorporation [103]. Several *in vitro* studies also obtained contradictory results in the influence of norepinephrine in *Salmonella* adherence, growth, and biofilm formation [102]. The first study found that norepinephrine increased biofilm formation of *S*. Enteritidis at 12 °C. However, this temperature does not represent the temperature at which *Salmonella* is found in the host. The second study [104] reported that *S*. Heidelberg adhesion and growth were not influenced by the presence of norepinephrine in the growth media. It should be considered that not all *Salmonella* serotypes and strains may respond similarly to the presence of catecholamines, and it is possible that serotypes with a higher virulence, such as *S*. Typhimurium and *S*. Enteritidis, have more efficient mechanisms to respond to the presence of catecholamines. Finally, in another innovative study, Reiske et al. [105] evaluated the immunomodulatory role of *S*. Typhimurium supernatant growth in the presence of catecholamines. The authors observed that supernatants reduced lymphocyte proliferation in a dose-dependent manner, probably because of the presence of immunomodulating substances. This research demonstrated the interkingdom cross-talk and the influence of stress on the invasiveness of pathogens such as *Salmonella*.

## 5. Conclusions

Bacterial cells cannot be considered as individual entities with individual functions. Both in the environment and in the host, bacterial cells of the same or different species act together in a coordinated manner to achieve a common goal. *Salmonella* cells communicate with each other through different mechanisms, such as quorum sensing, which is of great importance for the survival of *Salmonella* in different environments. *Salmonella* can respond to the presence of quorum sensing molecules produced by other bacterial groups and modulate its behavior in food to favor its survival. Also, *Salmonella* can use quorum sensing to modulate the gut microbiota and increase the probability of colonizing the host's gut. This food-borne pathogen is even capable of communicating with the host, using a quorum sensing mechanism about which much remains to be discovered, the AI-3. Through this mechanism, *Salmonella* can detect stress molecules, activate the expression of virulence factors, and increase its colonization success. Due to the importance of this mechanism, it is necessary to develop new methods that allow its intervention as a new tool to fight food pathogens.
